# The role of environmental exposure to non-cigarette smoke in lung disease

**DOI:** 10.1186/s40169-018-0217-2

**Published:** 2018-12-05

**Authors:** Rajendra KC, Shakti D. Shukla, Sanjay S. Gautam, Philip M. Hansbro, Ronan F. O’Toole

**Affiliations:** 1 School of Medicine, College of Health and Medicine, University of Tasmania, Hobart, Tasmania Australia; 2 Department of Clinical Microbiology, School of Medicine, Trinity College Dublin, Dublin, Ireland; 3 Priority Research Centre for Healthy Lungs, Hunter Medical Research Institute, School of Biomedical Sciences and Pharmacy, University of Newcastle, Newcastle, Australia; 4 Centenary Institute and University of Technology Sydney, Sydney, New South Wales Australia

**Keywords:** Non-cigarette smoke, Biomass smoke, Occupational exposure, Air pollution, Lung disease, Chronic obstructive pulmonary disease (COPD)

## Abstract

Chronic exposure to household indoor smoke and outdoor air pollution is a major contributor to global morbidity and mortality. The majority of these deaths occur in low and middle‐income countries. Children, women, the elderly and people with underlying chronic conditions are most affected. In addition to reduced lung function, children exposed to biomass smoke have an increased risk of developing lower respiratory tract infections and asthma-related symptoms. In adults, chronic exposure to biomass smoke, ambient air pollution, and opportunistic exposure to fumes and dust are associated with an increased risk of developing chronic bronchitis, chronic obstructive pulmonary disease (COPD), lung cancer and respiratory infections, including tuberculosis. Here, we review the evidence of prevalence of COPD in people exposed to non-cigarette smoke. We highlight mechanisms that are likely involved in biomass-smoke exposure-related COPD and other lung diseases. Finally, we summarize the potential preventive and therapeutic strategies for management of COPD induced by non-cigarette smoke exposure.

## Introduction

Worldwide, in 2016, approximately 9 million deaths were attributed to lung diseases, including COPD, lung cancer and lower respiratory tract infections, among which 82.4% occurred in low and middle-income countries [1]. Tobacco smoking is one of the most well-characterized risk factors for lung disease development, killing more than 7 million people each year [2]. However, compared to 1.1 billion tobacco smokers globally, nearly 3 billion people are exposed to biomass smoke [3, 4]. Moreover, data from epidemiologic studies conducted in Asia, Europe, South America and Africa have consistently shown associations between biomass smoke exposure and lung diseases, even after controlling for the primary risk factor, tobacco smoking [5, 6]. Here, we review the association of lung diseases, including COPD, with exposure to biomass smoke, fumes, dust, gases and outdoor air pollution.

## Methods

We conducted a qualitative examination of the published peer-reviewed literature from 1980 to June 2018 using the Medline database (https://www.nlm.nih.gov/bsd/medline.html). We used the search terms “biomass fuel”, “biomass smoke”, “wood smoke”, “solid fuel”, “coal smoke”, “indoor smoke”, household pollutants”, “outdoor air pollution”, “occupational exposure” combined using the Boolean logic ‘OR’. Similarly, the search terms “lung disease”, “pulmonary disease”, “airway disease”, “bronchitis”, “emphysema”, “asthma”, “lung cancer”, “acute respiratory tract infection” were also combined using the Boolean logic ‘OR’. The two subsets were combined using the Boolean logic ‘AND’, and the results were restricted to meta-analyses, systematic reviews, observational studies, comparative studies and original articles published in English. The search resulted in 1953 articles that were screened from which 168 studies were selected as being relevant to the review.

## Prevalence and burden of biomass smoke exposure

**Fig. 1:**
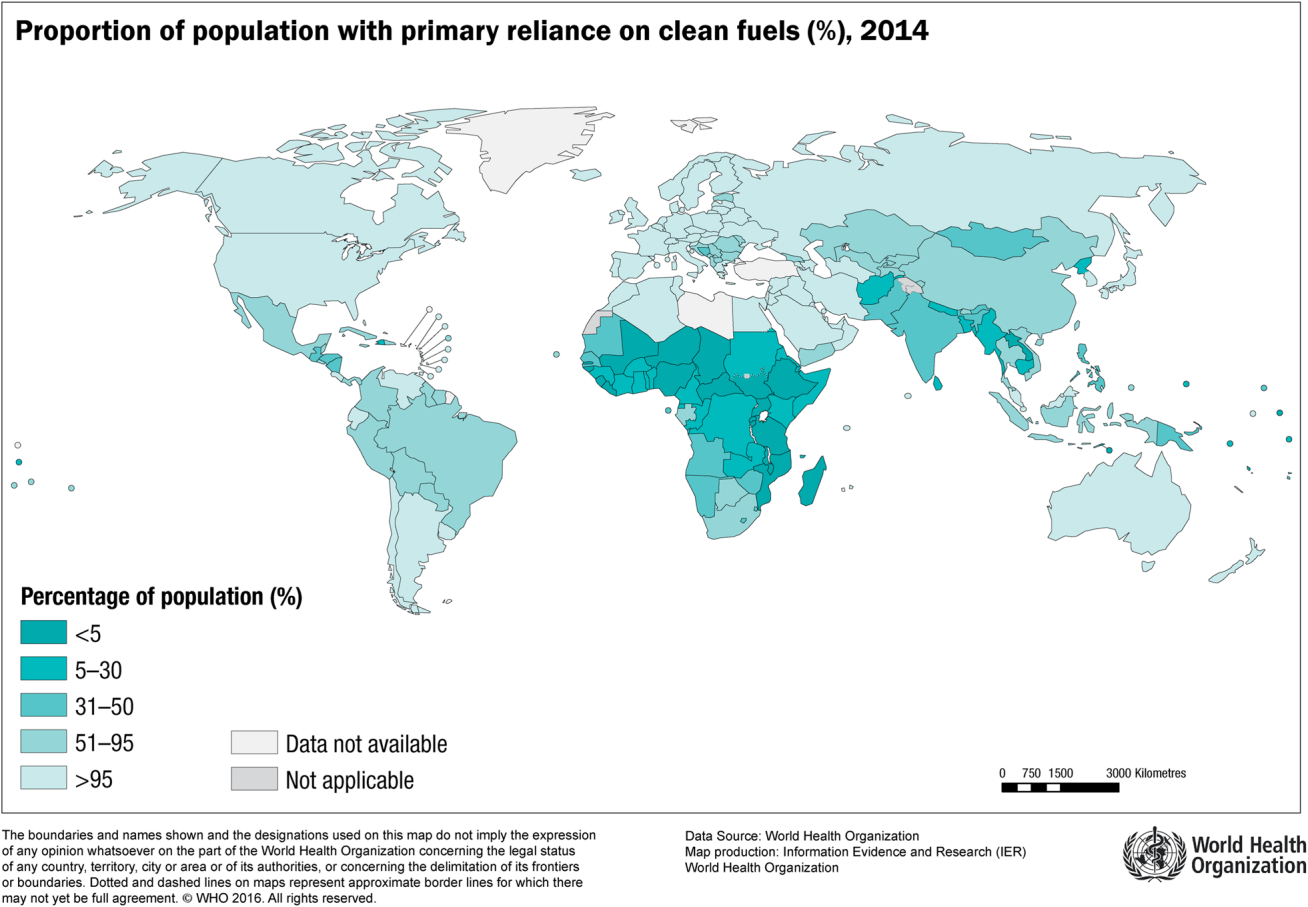
Global use of clean fuels in 2014, by the World Health Organization. Countries with the lowest (< 5%) and the highest (> 95%) proportion of people using clean fuels as the primary domestic source of energy are shaded dark and light blue, respectively. Reproduced with permission from the World Health Organization [8], Copyright (2016)

Annually, nearly 4 million people die prematurely from illness attributable to household biomass smoke exposure, among which an estimated 55% die due to respiratory diseases, including pneumonia (lower respiratory tract infections), COPD and lung cancer [7]. Nearly half of the world’s population rely on biomass, such as wood, animal dung, and crop residues as a primary fuel source for cooking and heating purposes [7]. The proportion of households using clean fuels (liquid petroleum gas, biogas and electricity) varies considerably across the globe (and even in the same continent) [8]. The access to clean fuels is especially limited to relatively smaller populations in the low- and middle-income countries (Fig. 1). Consequently, populations residing in the regional and rural households in developing countries are heavily reliant on biomass fuel for cooking or heating purposes (Fig. 1) [9].

The particular concern with energy from biomass fuel is the use of inefficient stoves for combustion, which generates toxic gases like carbon monoxide and nitrogen oxides; suspended particulate matter containing volatile organic compounds (VOCs) such as methane, aldehydes, benzene and its derivatives; and polycyclic aromatic hydrocarbons (PAHs) like benzo[*a*]pyrene and anthracene [10]. Particulate matter (PM) with an aerodynamic diameter of < 2.5 microns (PM_2.5_) is light and can remain suspended in the air for longer periods [11]. These particles can be inhaled deep into the lungs, and have been linked to oxidative stress and inflammation induced damage of the respiratory system [11]. Moreover, in developing countries, the cultural practice of indoor cooking in housing with poor air ventilation potentially exposes women and children to PM_2.5_ up to the levels that are 1000 times higher than the threshold recommended by WHO (25 µg/m^3^) [12, 13].

Wood is the most common biomass fuel, however, use of animal dung, such as cow, sheep and horse, as a source of fuel is also widespread, especially in rural areas of low- and middle-income countries, including India, Nepal, and sub-Saharan Africa due to its availability in areas with limited vegetation and its lower cost [14]. Despite this, animal dung is the least efficient biofuel and burns faster as compared to wood [15]. Relative to wood smoke, combustion of animal dung produces more particulate matter (23% more PM_2.5_ per kilogram), toxic byproducts, such as PAHs and oxidizing species, such as redox active metals (copper and iron) and quinones [16, 17].

Nonetheless, exposure to biomass smoke is not exclusively an issue in low- and middle-income countries. Use of indoor wood fires for heating purposes and for imparting flavor during cooking processes, such as barbecuing and wood-smoking of food, is becoming more popular in high-income countries, thus, increasing biomass smoke exposure [18]. In 2014, in a survey carried out in Australia, approximately 10% of households used wood as the main source of heating [19]. However, in developed countries, use of biomass fuel is primarily seasonal and exposure is largely limited by better ventilation [20]. Besides indoor biomass smoke exposure, people in developed countries such as Australia, Canada and the USA are also exposed to outdoor biomass smoke from frequent bushfires [21]. Importantly, individuals exposed to biomass smoke are more likely to have respiratory symptoms and reduced lung function [22–24].

## Association of biomass smoke exposure with respiratory diseases

### Chronic airway disease

Worldwide, COPD claimed more than 3 million deaths in 2016, among which 81.8% occurred in low- and middle-income countries [1]. Tobacco smoking is still the main causative factor of COPD in high-income countries where only 15–25% of COPD cases are never smokers (Table 1). However, in low and middle countries more than 45% of COPD patients have been found to be never smokers (Table 1). The existing literature provides strong evidence that smoke from biomass fuels is an independent risk factor for the development of COPD, particularly in low- and middle-income countries where the reliance on biomass fuel is still very high. The noxious particles in biomass smoke induce an inflammatory response through: upregulation of pro-inflammatory cytokines such as interleukin 6 (IL-6), tumor necrosis factor alpha (TNFα), and granulocyte colony stimulatory factor (G-CSF); recruitment of immune cells, such as macrophages and neutrophils; upregulation of gelatinases (matrix metalloproteinase 2 and 9); and epithelial-mesenchymal transition (EMT), thereby reducing lung function and contributing to the onset/progression of COPD [25–27]. Da Silva et al. in a case–control study in Brazil, evaluated the effect of exposure to biomass combustion PM_2.5_ on lung function [22]. There was a significant loss in pulmonary function in non-smoker biomass users compared to non-smoker liquefied petroleum gas (LPG) users (forced expiratory volume in one second to forced vital capacity ratio (FEV_1_/FVC) 0.79 versus 0.85, *p* < 0.05, respectively). In addition, pulmonary function was negatively correlated with the level and duration of PM_2.5_ exposure (FEV_1_/FVC: r = − 0.63, *p* < 0.05 and − 0.52, *p *< 0.05, respectively) [22]. A minor reduction in FEV_1_/FVC was observed in women in Mexico using biomass fuel compared to clean gas (0.80 vs 0.83, *p* = 0.03, respectively) [24].

**Table 1:** Proportion of never smokers among COPD patients and associated risk factors

Study center and design	Participants	Age (years)	Proportion of never smokers among COPD patients (%)			Risk factors for COPD in never-smokers	References
			Overall	Male	Female		
Multinational (35 centres, 16 countries; ECRHS)	17,966	20–44	17.0	13.4	21.6	Occupational exposure to vapours, gas, dust, or fumes	Cerveri et al. [33]
Malataya, Turkey (CS)	1160	> 18	22.5			Exposure to biomass smoke	Gunen et al. [34]
China (CPH; nationwide CS)	50,991	> 20	50.5	10.1	91.7	Exposure to biomass fuel smoke and PM_2.5_, parental history of respiratory disease	Wang et al. [28]
South Africa (nationwide survey)	13,826	> 18	47·6	24.8	61.0	Biomass fuel, occupational exposure, history of pulmonary tuberculosis	Ehrlich et al. [35]
Maswa, Tanzania (CS)	869	> 35	62.1	–	–		Magitta et al. [36]
Västra Götaland and Norrbotten, Sweden (CS)	1839	21–78	21	–	–	Occupational exposure to gas, dust and fumes	Hagstad et al. [37]
Copenhegen, Denmark (CS)	68,501	20–100	22.3	18.9	25.5		Thomsen et al. [38]
Multinational 12 countries; population-based survey)	73,745	> 40	36 (24 USA-64 Mexico)	21	49		Landis et al. [39]

*ECRHS* European Community Respiratory Health Survey; *CS* cross-sectional study; *CPH* China Pulmonary Health; *COPD* chronic obstructive pulmonary disease

In a recent nationwide cross-sectional study conducted in 50,991 individuals from ten provinces of China, more than half of the COPD patients were never smokers [28]. Importantly, the proportion of never smokers in female COPD patients was markedly high (91.7%). Additionally, individuals exposed to indoor biomass smoke were 62% more likely to develop COPD than unexposed individuals who used clean fuel sources (age and sex adjusted OR = 1.6, *p* = 0.003) [28].

A meta-analysis of 11 cross-sectional and four case–control studies covering a wide range of countries identified household biomass smoke exposure as an independent risk factor for developing COPD in both men (OR 4.30, 95% CI 1.85–10.01) and women (OR 2.73, 95% CI 2.28–3.28), and in both the Asian population (OR 2.31, 95% CI 1.41–3.78) and the non-Asian population (OR 2.56, 95% CI 1.71–3.83) [5]. This finding is supported by another recent cross-sectional study, the PUMA (Prevalence Study and Regular Practice, Diagnosis, and Treatment Among General Practitioners in Populations at Risk of COPD in Latin America), which assessed 1740 individuals from multiple nations in South America, who were at greater risk of developing COPD [6]. The PUMA study reported that individuals exposed to household biomass smoke are twice as likely to develop COPD than unexposed people (adjusted OR 2.28, 95% CI 1.18–4.41) [6]. Moreover, a systematic review of 24 epidemiological studies revealed that household biomass smoke exposure was associated with COPD development in both urban (OR 1.6, 95% CI 1.2–2.0) and rural women (OR 2.0, 95% CI 1.5–2.8) [29].

In terms of COPD phenotypes, biomass smoke exposed individuals were found to have more lung fibrosis and bronchiolitis and less emphysema whereas tobacco smokers exhibited higher levels of emphysema (radiologist CT score—a measure of the extent of emphysema based on computed tomography (CT) at inspiration and expiration) 0.7 versus 2.3, *p *= 0.001; emphysema on CT 19% versus 27%, *p *= 0.046) [30, 31]. This finding is further supported by Fernandes et al. who reported a significantly lower level of emphysema in biomass exposed women than in women who were tobacco smokers (PRM^Emph^ (parametric response mapping, an image tool used to quantify emphysema based on paired CT images at inspiration and expiration) 1.84% (0.69 ± 3.72%) versus 9.85% (2.40–16.34%); *p* = 0.001) [32].

### Lung cancer

Globally, 1.7 million deaths in 2016 were attributed to lung cancer, among which 66% occurred in low- and middle-income countries [1]. The combustion products of biomass fuel emissions such as dibenz[*a,h*]anthracene, cyclopenta[*cd*]pyrene and 1,3-butadiene have been grouped as a probable human carcinogen (group 2A) by the International Agency for Research on Cancer [40]. Saldana and colleagues, in a hospital based case–control study with 136 cases of primary lung cancer and 137 controls, reported a significant association between the magnitude of biomass smoke exposure in hour-years (years of exposure multiplied by average hours of exposure per day) and the risk of lung cancer after adjusting for sex, smoking, socioeconomic status and housing with asbestos sheet roof (OR for an exposure > 300 h-years 3.01, 95% CI 1.12–8.36) [41]. A systematic review including 13 case control studies summarized an increased risk (OR 1.17, 95% CI 1.01–1.37) of lung cancer with biomass exposure associated with cooking/heating, in particular, among women in developing countries [42]. In a retrospective cohort study conducted in Xuanwei city of China, mortality from lung cancer was compared between lifelong users of smoky coal (27,310 individuals) and smokeless coal (9962 individuals). Individuals exposed to smoky coal were at greater risk of death from lung cancer when compared to those exposed to smokeless coal (for men, HR 36, 95% CI 20–65; for women, 99, 95% CI 37–266) [43]. A similar observation was reported in a meta-analysis from 28 epidemiological studies where a significantly higher risk of lung cancer was observed in females (OR 1.81, 95% CI 1.54–2.12; *p* = 0.034) exposed to biomass/solid fuel smoke compared to males (OR 1.16, 95% CI 0.79–1.69) with related exposure [44]. The increased susceptibility of women to biomass/coal carcinogens compared to men may be due to differences in base-line exposure (women being exposed to biomass/coal smoke for extended periods of time) or due to differences in lung size (women having smaller lungs than men, hence more damage done for the similar amount of smoke inhaled) [45]. The association was even more prominent in individuals using coal (OR 1.82, 95% CI 1.60–2.06) compared to biomass (OR 1.50, 95% CI 1.17–1.94), which may be due to the production of predominant Group 1 carcinogenic PAHs during coal combustion [44, 46]. Although the studies included in this review have either been adjusted for smoking or studied a population of non-smokers, none of these studies commented on the presence of other co-morbid conditions, such as COPD, at the time of lung cancer diagnosis.

### Lower respiratory tract infections

In addition to COPD and lung cancer, the other major respiratory cause of global mortality is lower respiratory tract infection (LRI) and tuberculosis, which was directly implicated in 2.96 and 1.29 million deaths in 2016, respectively [1]. Of the approximate 4 million deaths globally, around 89% of the mortality was reported in low- and middle-income countries alone [47]. An evaluation of 14 studies in developing countries indicates that young children living in households where biomass fuels are utilized, have a two to three times higher risk of respiratory infections than unexposed children after adjustment of potential confounding factors [48, 49]. A recent systematic review of 77 studies from 39 low- and middle-income countries evaluated the risk factors for mortality from acute lower respiratory infections in children under 5 years of age and found that biomass smoke exposure is significantly associated with an increased risk of death from LRI (OR 3.0, 95% CI 2.1–4.3) [50]. However, only a small number of studies have focused on the association between biomass smoke exposure and risk of lower respiratory tract infection in the adult population. Ezzati et al. evaluated the risk of LRI in 229 individuals between 5 and 49 years of age in Central Kenya upon exposure to biomass fuel derived PM_10_ [51]. The risk of LRI was positively correlated with the level of PM_10_ exposure, however, the odds ratio was significant only above 2000 µg/m^3^ (adjusted OR 3.3, 95% CI 1.1–9.9) [51]. Exposure to household biomass smoke was associated with risk of hospitalization due to pneumonia in older adults aged 65 and above (adjusted OR 3.3, 95% CI 1.6–6.9) [52]. On the other hand, although the prevalence of acute respiratory infection was higher among biomass users (15–45%), after controlling for major confounding factors such as age and smoking, the risk of LRI was not significant when including adults aged less than 65 [53–55]. In addition, biomass smoke exposure is significantly associated with the risk of developing tuberculosis. For example, a close correlation between biomass fuel usage and tuberculosis (OR 3.14, 95% CI 1.15–8.56) has been reported from northern India compared to usage of liquid petroleum gas in kitchens after adjusting for confounding variables, such as tobacco smoking and close contact with TB cases (6).

### Other respiratory conditions

Several studies have highlighted the effect of biomass smoke exposure in respiratory symptoms, such as cough, wheeze, mucus overproduction and dyspnoea [22–24]. A case–control study in Brazil reported an increased risk of developing chronic cough (OR 2.9, 95% CI 1.68–5.10), wheeze (OR 2.33, 95% CI 1.25–4.38) and dyspnoea (OR 2.59, 95% CI 1.32–5.09) in adults exposed to biomass smoke as compared to LPG users [22]. Similarly, a study in Eastern India assessed the impact of chronic exposure to indoor biomass smoke on respiratory health in 681 non-smoking women and 438 age matched women not exposed to biomass smoke [23]. Compared to LPG users, biomass users had a higher prevalence of cough, mucus production and wheeze (71.8% versus 30.8%, *p* = 0.001), and dyspnoea (58.4% versus 19.9%, *p* = 0.001) [23].

Besides lower respiratory tract symptoms, other lung diseases, such as asthma and pneumoconiosis have been correlated with exposure to indoor biomass smoke. Oluwole et al. reported an increased prevalence of asthma-related symptoms, such as wheezing or whistling in the chest, cough, dyspnoea and chest tightness in rural school children in Nigeria exposed to biomass smoke (OR 2.37, 95% CI 1.16–4.84) [56]. However, evidence of its association with asthma is inconclusive. A meta-analysis of 25 studies did not find any significant association of biomass smoke exposure and asthma in children (OR 0.5, 95% CI 0.1–2.0) or women (1.34, 95% CI 0.9–1.9) [57]. However, a significant difference has been reported between bronchiectasis (diagnosed by computed tomography scans) in women and exposure to biomass fuel, compared to exposure to tobacco smoke (14% versus 0%, *p* = 0.009) [30]. Particularly, the “Hut lung disease” or domestically acquired pneumoconiosis has been noted as a particulate lung disease in women exposed to biomass fuel smoke or agricultural activities but not associated with mining [58–60].

## Exposure to other types of air pollutants

### Environmental exposure to noxious particles and gases

High levels of air pollution have also been implicated as risk factors for the development of COPD, although it is less potent than active smoking. Approximately 25–60% of COPD patients are never-smokers in developing countries [28, 36, 61]. Increasing population, urbanization, economic profile and pollution are several factors that contribute substantially to the COPD burden [62]. The role of outdoor air pollution (including traffic-related fine particulate matter) as a causative factor for airflow limitation is gaining attention in recent times, due to increased vehicular pollution, as well as industrialization of the two most populated countries, China and India [63].

PM_2.5_ is considered to be the most health-damaging of the particulate matter, as it can penetrate deep into the lungs and initiate deleterious effects on the airway, including but not limited to airway oxidative stress, pulmonary and systemic inflammation, ciliary dysfunction, amplification of infections, and increases in bronchial reactivity [64]. The main components of PM_10_ are sulfate, nitrates, ammonia, sodium chloride, black carbon, mineral dust and water [4].

Several investigators have assessed the effect of air pollution as a potential risk factor for COPD. In children and young adults, cross-sectional studies have shown a relationship between higher outdoor pollutant levels (especially traffic related pollution) and lower lung function [65, 66]. Kulkarni et al. reported a likely causative dose-dependent inverse association between the carbon content (as a biomarker of particulate matter exposure, PM_10_) of airway macrophages with lung function in children [67]. Furthermore, exposure to traffic-related pollution, indicated by residential distance from a highway, was associated with impaired lung growth and lung function deficits at 18 years of age [68]. Also, higher traffic density and proximity to highways was significantly associated with lower lung function (FEV_1_) and FVC, but only in females [69], who are also at a greater risk of developing COPD than for those living farther away [70].

Long-term exposure to airborne particles and particulate matter is significantly associated with the risk of premature death and acute care hospitalizations, especially in patients with severe disease [71, 72]. Moreover, daily variation in exposure to outdoor air pollution (mainly the particulate matter) significantly correlates with acute exacerbations of COPD [73]. The mechanisms that underlie obstruction due to air pollution are likely to be similar to those caused by cigarette smoking, but we do not yet have conclusive evidence for this, despite the detrimental effects of air pollution on lung health.

### Occupational exposure to dusts and fumes

Several longitudinal studies have shown an association between certain dusty occupational exposures and COPD, i.e., coal mining [74], gold mining [75], work related to tunnel-construction [76], low levels of concrete dust containing crystalline silica exposure (concrete production industries) [77], exposure to cotton in textile industries [78], workers exposed to welding fumes [79], grain handlers and postal workers (exposure to endotoxins) [80] and animal feed industry [81]. Moreover, chronic exposure to metallic dust (primarily, cobalt and chromium) was found to be associated with deterioration of lung function [82]. In addition, exposure to chemical vapors, irritants and fumes can also contribute to accelerated loss of airflow [83]. Another study involving railroad workers reported a positive association between COPD mortality and occupational exposure to diesel exhaust [84]. A population-based study found positive associations between several occupational exposure measures (mineral dusts, metal dusts and fumes, organic dusts, irritant gases or vapors, sensitizers, organic solvents, diesel exhaust, and environmental tobacco smoke) with COPD, among both ever-smokers and never-smokers [85].

Experimental studies in animal models have demonstrated that exposures to several agents, such as sulphur dioxide, mineral dusts, vanadium and endotoxin, are capable of inducing chronic obstructive bronchitis [83]. Intratracheally instilled silica (quartz) produces airflow obstruction (functional change), which correlates with the presence of both emphysema and small-airway lesions [86]. Inorganic dusts containing silica are also associated with neutrophil and macrophage accumulation and morphological changes in the rat lung [86]. These morphological changes in small airways and lung parenchyma were similar to those in rats treated with elastase, which represents a well-established model of experimental COPD [87].

## Mechanisms of lung damage due to biomass smoke exposure

Despite the immense burden of chronic airway disease, only a limited number of mechanistic studies have examined the immunomodulatory effects of biomass smoke exposure. Sussan et al. explored the mechanisms of pulmonary responses in mice after acute (6 and 24 h) or sub-chronic (3 times a week for 8 weeks) exposure to wood or cow dung PM [17]. Acute exposure to wood smoke elicited a stronger pro-inflammatory response, as indicated by increased expression of G-CSF, Keratinocyte chemoattractant (KC), C-X-C motif chemokine 10 (CXCL10), IL-6, TNFα and interleukin 12 p70 subunit (IL12p70) [17]. The induction of pro-inflammatory cytokine expression was higher with cow dung than wood smoke PM [17]. On the other hand, sub-chronic exposures exhibited differences in pulmonary response, where wood smoke elicited an eosinophilic response in contrast to a predominantly neutrophilic response induced by cow dung smoke [17]. The inflammatory response elicited by both wood and cow dung smoke was mediated via nuclear factor kappa B (NF-κB) signaling [17]. In addition, in an in vitro study, c-Jun N-terminal kinase-activator protein-1 (JNK-AP-1) signaling, and not the NFκB regulatory pathway, was found to be involved in mediating inflammatory responses in human primary small airway epithelial cells upon exposure to dung biomass smoke [88].

Moreover, in a case–control study conducted in India with 142 biomass users and 126 age-matched LPG user women, a significant neutrophilic inflammatory response was observed in the biomass using group [89]. Also, compared to LPG users, reactive oxygen species (ROS) generation by leukocytes (in both blood and sputum) and the systemic level of antioxidant enzyme superoxide dismutase (SOD) were higher and lower, respectively in women using biomass fuel [89]. A similar increase in systemic oxidative stress was reported in rats exposed to biomass smoke, as indicated by an increased plasma level of malondialdehyde and a reduced level of SOD [90]. Several in vitro studies reported depletion of antioxidants, such as ascorbate, urate and reduced glutathione in respiratory tract lining fluid when incubated with wood and animal dung smoke extracts [16, 91]. Therefore, biomass smoke exposure induces inflammation and oxidative stress mediated lung damage which potentially contributes to the development/progression of COPD.

In terms of lung cancer, biomass smoke induced ROS directly damages DNA and promotes lung cell proliferation and turnover, resulting in fibrosis and development of lung tumours in rats [92]. Furthermore, biomass exposure upregulates the production of extracellular matrix proteins, including perlecan and fibronectin with a capacity to induce fibrosis in cultured cells [93]. In addition, PAHs released by incomplete combustion of biomass fuels are evidently carcinogenic in both in vitro and in vivo studies [94–97]. Metabolism of PAHs leads to the formation of active carcinogens diol-epoxides, radical cations and o-quinones [94, 98]. These activated PAH metabolites can form adducts with DNA, resulting in mutations, alteration of gene expression profiles, and tumorigenesis [94]. Gene ‘hotspots’ for adduct formation by PAH metabolites include oncogenes such as p53, K-ras and the H-ras [99, 100]. PAH-DNA adducts are associated with a two-fold increased risk of lung cancer [101].

Biomass smoke exposure is also implicated in increased susceptibility to bacterial infection through several mechanisms, including alterations in alveolar macrophage phagocytosis and/or upregulation of host surface receptors on the respiratory epithelium [102, 103]. In an in vitro study, the phagosomal function of wood smoke particle exposed human alveolar macrophages was tested with respect to uptake of fluorescently-labelled beads, *Streptococcus pneumoniae* and *Mycobacterium tuberculosis* [102]. Wood smoke exposed macrophages demonstrated reduced phagocytosis of fluorescent beads, *S. pneumoniae* and *M. tuberculosis* with a negative linear correlation between macrophage particulate content and phagocytosis [104]. Furthermore, oxidative stress is believed to upregulate expression of intercellular adhesion molecule-1 (ICAM-1), and platelet activating factor receptors (PAFR) allowing attachment and invasion of respiratory bacteria, including *S. pneumoniae*, *Haemophilus influenzae* and *Pseudomonas aeruginosa,* which are also important bacterial pathogens in COPD [105–107]. Although airway ICAM-1 and PAFR expression were markedly upregulated in tobacco smokers and COPD patients, further studies are warranted to demonstrate the effect of biomass smoke exposure on inducing the expression of these host surface receptors [108–110]. Recently, KC et al. developed a method to generate batches of biomass smoke extracts that can be preserved for longer periods for use in multiple exposure experiments, thus minimizing inter-assay variation [103]. This will facilitate further research on mechanistic role of smoke extracts in the inflammatory response and the pathogenesis of respiratory diseases including COPD, lung cancer and LRI.

## Preventive and therapeutic measures

Biomass smoke is one of the major risk factors for the onset/progression of COPD in developing countries [61]. To reduce the exposure to smoke generated from combustion of biomass fuel, several preventive interventions have been recommended, with some already implemented.

Based on previous intervention studies, the use of efficient cooking stoves and the promotion of cleaner fuel use (e.g., biogas, liquefied petroleum gas and electricity) has been effective in preventing biomass smoke exposure, at least to some extent [111–113]. The use of improved cooking stoves (ICS) has been found to reduce the mean PM_2.5_ levels by 63.2% in Nepal, 37.0% Senegal, 32.8% in the Gambia and 18–45% in Kenya [111, 112, 114]. Although the decrease in mean PM_2.5_ levels was significantly high, none of the improved cooking stoves achieved the WHO guideline level of mean kitchen PM_2.5_ of 25 µg/m^3^ for 24-h. A recent 9-year prospective cohort study in China evaluated the effect of intervention with improved cooking fuels and kitchen intervention [113]. Use of clean fuels and improved ventilation were associated with a lower decline in FEV_1_ (12 mL/year, 4–20 mL/year versus 13 mL/year, 4–23 mL/year) compared to those with neither intervention, after adjusting for confounders [113]. In addition, the duration of improved fuel use and ventilation was negatively correlated with the decline of FEV_1_ (*p* < 0.05) [113]. Moreover, the fuel and ventilation improvement intervention were associated with a reduction in the risk of COPD, with an odds ratio of 0.28 (95% CI, 0.11–0.73) [113]. Despite the perception that biomass is a cheap fuel source, the annual health-related cost per household associated with biomass smoke exposure (16.94 USD) in Nepal is 61.3% higher than the annual cost of biogas usage (10.38 USD), an alternative cleaner fuel [14]. In addition, the findings of the study suggest that providing community education and creating rural employment and income generation opportunities are important measures in promoting the sustainable use of clean fuels [14].

In terms of therapeutic intervention, exposure to household and outdoor air pollutants are found to reduce antioxidant defense by decreasing the levels of ascorbate, urate, SOD and reduced glutathione resulting in inflammation of the airways [115]. Antioxidants and anti-inflammatory drugs are potential therapeutics for the prevention of biomass smoke induced inflammation-mediated lung injury [116, 117]. Based on the evidence that wood and cow dung smoke upregulated the expression of PAFR on human bronchial epithelial cells, we believe that the induction of microbial adhesion receptors on respiratory epithelia could be targeted therapeutically to prevent LRIs. Several in vitro studies have shown that the use of PAFR antagonists such as CV-3988 and WEB-2086 abrogates the adhesion of *S. pneumoniae* and nontypeable *H. influenzae* to cigarette smoke exposed human bronchial and alveolar epithelial cells [118, 119]. In addition, ICAM-1, a major receptor for rhinoviruses and nontypeable *H. influenzae* could be another important target with therapeutic potential [120, 121]. However, we stress the point that further research is needed to elucidate the mechanisms of biomass smoke induced susceptibility to LRI, as well as development of COPD and lung cancer. But while new therapeutics may be possible with regard to reducing the progression or symptoms of biomass smoke-related lung disease, the major focus needs to be maintained on a global reduction in the exposure of human populations to biomass smoke.

## Strengths and limitations

There are several strengths of our review. Our narrative review comes at a time when the United Nations and the World Health Organization have specifically focused on the urgent need to limit non-communicable diseases, which has also garnered support from various Heads-of-Countries and Governments [122]. In addition, we have examined the epidemiology of non-cigarette smoke etiologies (biomass smoke, air pollution and multiple occupational exposures) implicated in heightened risk of developing a range of lung diseases, most notably, COPD, lung cancer and respiratory infections. We have reviewed and summarized potential mechanisms involved in the pathogenesis of non-cigarette smoke exposure and lung disease. Finally, we have highlighted the potential preventive, as well as therapeutic strategies to limit lung diseases related to these exposures.

One of the limitations is that there may be variability across epidemiological studies in terms of data collection and analyses. In particular, factors such as geographical locations, sampling points within a city as well as multiple cities in a specific country, the type of sampling (longitudinal versus one time-point sampling) contributes significantly to heterogeneity of results obtained. In addition, participant recruitment criteria (i.e., from general population or community healthcare centers), reliability of self-reported duration of exposure are crucial factors that need to be standardized globally. Notably, variability of spirometry technique and the threshold cut-off for COPD (i.e., FEV_1_/FVC < 0.70 or FEV_1_/FVC < LLN) vary across studies. In addition, it is difficult to tease out contributions of individual exposure(s)-related causations and hence the results may represent disease associated with mixed exposures. The heterogeneity in future epidemiological studies could be addressed by utilizing standardized and validated research methodologies/strategies to ascertain burden of lung diseases associated with non-cigarette smoke exposures. We are not concluding direct causation of lung diseases due to non-cigarette smoke exposures in our review; however, our intent was to direct policymakers and researchers towards the possible mechanisms reported to date. Clearly, focused and detailed mechanistic studies are needed to establish direct causation of non-cigarette smoke exposures and lung pathologies, which should be further validated in large-scale epidemiological studies, as well as mechanistic models of experimental animal models. Finally, we limited our analysis to published literature and did not include media/analytical reports from recently initiated preventive strategies (such as Ujjwala yojana in India; [123]). However, this could be followed up once the government reports are released by various countries.

## Summary and conclusions

The prevalence of COPD amongst non-smokers is a worrying concern globally, as almost half the World’s population is chronically exposed to biomass smoke, air pollution and other exposures (chemicals, dusts, and fumes). Evidently, low socio-economic status drives large sections of societies living in low- and middle-income countries towards biomass fuels for cooking/heating purposes. In addition, poor awareness about respiratory health and lack of implementation of adequate safety measures (e.g., poorly ventilated homes) predispose these individuals to heightened risk of developing biomass smoke-induced lung diseases, including COPD, lung cancer and respiratory infections. Chronic biomass smoke (and other non-cigarette exposures) delivers deleterious compounds (PMs, PAHs etc.) that activate an inflammatory cascade in the lungs, which may potentially spill over systemically. This is coupled with marked immune cell dysfunction, activation of oncogenes and upregulation of microbial receptors in the respiratory tract, which contribute to onset/progression of COPD and other related diseases (lung cancer and infections). Preventive strategies should include awareness about the long-term health benefits of using cleaner fuels over biomass-fuels, use of more efficient cooking equipment, and the importance of adequate ventilation in low socioeconomic localities. Although research has only begun to unravel the underlying molecular and pathological mechanisms involved in biomass smoke-induced COPD, anti-oxidants, anti-inflammatory and anti-microbial drugs may be promising new therapeutics to limit the effects of non-smoking related COPD.

## Abbreviations

AP-1: activator protein 1
CI: confidence interval
COPD: chronic obstructive pulmonary disease
CS: cross-sectional study
CPH: China Pulmonary Health
CT: computed tomography
CXCL10: C-X-C motif chemokine 10
DNA: deoxyribonucleic acid
ECRHS: European Community Respiratory Health Survey
FEV_1_: forced expiratory volume in one second
FVC: forced vital capacity
OR: odds ratio
G-CSF: granulocyte colony-stimulating factor
HR: hazard ratio
ICAM-1: intercellular adhesion molecule-1
ICS: improved cook stoves
IL-12p70: interleukin 12 p70 subunit
IL-6: interleukin 6
JNK: c-Jun N-terminal kinase
KC: keratinocyte chemoattractant
LPG: liquefied petroleum gas
LRI: lower respiratory tract infection
NFkB: nuclear factor kappa B
PAFR: platelet activating factor receptor
PAHs: polycyclic aromatic hydrocarbons
PM: particulate matter
PRM: parametric response mapping
PUMA: Prevalence Study and Regular Practice, Diagnosis, and Treatment Among General Practitioners in Populations at Risk of COPD in Latin America
ROS: reactive oxygen species
SOD: superoxide dismutase
TB: tuberculosis
TNFα: tumour necrosis factor alpha
VOCS: volatile organic compounds
WHO: World Health Organization
